# Novel T7 Phage Display Library Detects Classifiers for Active Mycobacterium Tuberculosis Infection

**DOI:** 10.3390/v10070375

**Published:** 2018-07-19

**Authors:** Harvinder Talwar, Samer Najeeb Hanoudi, Sorin Draghici, Lobelia Samavati

**Affiliations:** 1 Department of Medicine, Division of Pulmonary, Critical Care and Sleep Medicine, Wayne State University School of Medicine and Detroit Medical Center, Detroit, MI 48201, USA; ar8673@wayne.edu; 2 Department of Computer Science, Wayne State University, Detroit, MI 48202, USA; ei1875@wayne.edu (S.N.H.); sorin@wayne.edu (S.D.); 3 Center for Molecular Medicine and Genetics, Wayne State University School of Medicine, 540 E. Canfield, Detroit, MI 48201, USA

**Keywords:** T7phage library, sarcoidosis, tuberculosis, microarray, immunoscreening

## Abstract

Tuberculosis (TB) is caused by *Mycobacterium tuberculosis* (MTB) and transmitted through inhalation of aerosolized droplets. Eighty-five percent of new TB cases occur in resource-limited countries in Asia and Africa and fewer than 40% of TB cases are diagnosed due to the lack of accurate and easy-to-use diagnostic assays. Currently, diagnosis relies on the demonstration of the bacterium in clinical specimens by serial sputum smear microscopy and culture. These methods lack sensitivity, are time consuming, expensive, and require trained personnel. An alternative approach is to develop an efficient immunoassay to detect antibodies reactive to MTB antigens in bodily fluids, such as serum. Sarcoidosis and TB have clinical and pathological similarities and sarcoidosis tissue has yielded MTB components. Using sarcoidosis tissue, we developed a T7 phage cDNA library and constructed a microarray platform. We immunoscreened our microarray platform with sera from healthy (*n* = 45), smear positive TB (*n* = 24), and sarcoidosis (*n* = 107) subjects. Using a student *t-*test, we identified 192 clones significantly differentially expressed between the three groups at a False Discovery Rate (FDR) <0.01. Among those clones, we selected the top ten most significant clones and validated them on independent test set. The area under receiver operating characteristics (ROC) for the top 10 significant clones was 1 with a sensitivity of 1 and a specificity of 1. Sequence analyses of informative phage inserts recognized as antigens by active TB sera may identify immunogenic antigens that could be used to develop therapeutic or prophylactic vaccines, as well as identify molecular targets for therapy.

## 1. Introduction

Tuberculosis (TB) remains a serious global health threat with 10 million new cases and 1.7 million deaths each year [1,2]. Currently, we have limited tools available to diagnose active TB, predict treatment efficacy and the cure of tuberculosis, or to detect the reactivation of a latent tuberculosis infection, and assay the induction of protective immune responses through vaccination. A major obstacle to global control of TB remains inadequate case detection [3]. Efforts during the past decade to consistently diagnose and treat most infectious cases have slowed the TB incidence rate, but have not yielded substantial progress [3]. The existing TB diagnostic pipeline still does not have a simple, rapid, inexpensive point-of-care test [3]. Qualified tuberculosis biomarkers are most urgently needed as predictors of reactivation and cure, and indicators of vaccine-induced protection [3].

Pulmonary tuberculosis has clinical and pathological similarities with sarcoidosis. Sarcoidosis is a systemic granulomatous disease of unknown etiology with predominant involvement of the lungs, among other organs [4,5,6,7]. Several studies have suggested that the cellular and humoral responses associated with granuloma formation in sarcoidosis are the consequence of an exaggerated immune response to specific *Mycobacterium tuberculosis* (MTB) antigens [4,8]. Sarcoidosis tissue has yielded MTB components including, ESAT6 and catalase—peroxidase (mKatG) [9]. Despite the presence of specific TB antigens in sarcoidosis lung tissues [8,10,11,12], patients with sarcoidosis negatively respond to the tuberculin skin test and are considered to be anergic [13]. Additionally, sarcoidosis subjects rarely ever develop tuberculosis. Lungs are highly involved both in sarcoidosis and TB. Resident alveolar macrophages (AMs) play an important role in the pathogenesis and host defense of both diseases [4,14,15,16]. It has been shown that AMs provide a reservoir for MTB and other slow growing organisms [11,14,15,17]. Additionally, AMs play an integral role in autoimmunity and the initiation of fibrosis [14]. Based on this knowledge, we hypothesized that bronchioalveolar cells (BALs) of sarcoidosis subjects may harbor degradation products of specific pathogen(s), including MTB. We constructed four T7 phage display cDNA libraries, two of which originate from sarcoidosis BAL cells and white blood cells (WBCs), and two others derived from cultured human embryonic fibroblasts and splenic monocytes, and combined all four libraries into a complex library [18,19]. We randomly selected 1070 clones through biopanning and constructed a microarray platform with the selected clones. Previously, upon immunoscreening of this platform with sera from healthy controls, sarcoidosis and culture positive TB patients, we showed that we can detect highly sensitive and specific biomarkers for TB in the sera of subjects with culture positive MTB [18,20]. In that study, the TB patients were smear negative but culture positive and at the time of sera collection, they were on treatment with anti-tuberculosis agents [18,20]. To investigate whether our display library also detects specific biomarkers in sera from smear positive MTB patients and if these biomarkers differ from those of smear negative but culture positive TB, we immunoscreened T7 phage display libraries with sera of smear-positive TB patients. The objective of the present study was to identify the specific diagnostic biomarkers from the sera of TB patients who had active TB. We discovered reactive clones that distinguished sera from active TB patients from sarcoidosis patients and uninfected control sera with a high sensitivity and specificity.

## 2. Materials and Methods

### 2.1. Chemicals

All chemicals were purchased from Sigma-Aldrich (St. Louis, MO, USA) unless specified otherwise. LeukoLOCK filters and RNAlater were purchased from Life Technologies (Grand Island, NY, USA). The RNeasy Midi kit was obtained from Qiagen, (Valencia, CA, USA). The T7 mouse monoclonal antibody was purchased from Novagen (San Diego, CA, USA). Alexa Fluor 647 goat anti-human IgG and Alex Fluor goat anti-mouse IgG antibodies were purchased from Life Technologies (Grand Island, NY, USA).

### 2.2. Patient Selection

This study was approved by the institutional review board at Wayne State University, and the Detroit Medical Center. Sera were collected from 3 groups: (1) healthy volunteers; (2) sarcoidosis subjects; and (3) smear positive pulmonary TB patients. All study subjects signed a written informed consent. All methods were performed in accordance with the human investigation guidelines and regulations by the IRB (protocol No = 055208MP4E) at Wayne State University. All sarcoidosis subjects were ambulatory patients. Sera from patients with tuberculosis were obtained from the Foundation for Innovative New Diagnostics (FIND, Geneva, Switzerland). All TB patients had smear positive sputum. 

### 2.3. Serum Collection

Using standardized phlebotomy procedures blood samples were collected and stored at −80 °C [18]. 

### 2.4. Construction and Biopanning of T7 Phage Display cDNA Libraries

We have used the same T7 phage display libraries as before [18,19]. Briefly, T7 phage display libraries from BALs, WBCs, EL-1 and MRC5 were made to generate a complex sarcoid library (CSL) [18,19]. Differential biopanning for negative selection was the performed using sera from healthy controls to remove the non-specific IgG, and sarcoidosis sera for positive enrichment as described previously [18,19].

### 2.5. Microarray Construction and Immunoscreening

A total of 1070 individually picked phage clones from the biopannings 3 and 4 for microarray construction were same as used in previous studies [18,19]. The phage lysates were arrayed in quintuplicates onto nitrocellulose FAST slides (Grace Biolabs, OR, USA) using the ProSys 5510TL robot (Cartesian Technologies, CA, USA). The nitrocellulose slides were hybridized with sera and processed as described previously [18].

### 2.6. Sequencing of Phage cDNA Clones

Individual phage clones were PCR amplified using T7 phage forward primer 5′ GTTCTATCCGCAACGTTATGG 3′ and reverse primer 5′ GGAGGAAAGTCGTTTTTTGGGG 3′ and sequenced by Genwiz (South Plainfield, NJ, USA), using T7 phage sequence primer TGCTAAGGACAACGTTATCGG. cDNA sequences of T7 phage clones obtained from Genwiz were translated into peptide/protein sequences using ExPASy *translate tool*. The length of each peptide clone is determined after the last amino acid of linker sequence (GDPNSS) inserted in frame of T7 phage till the stop codon of the sequence. Using NCBI protein BLAST site each identified sequence was used for further BLAST. For each peptide, we performed three BLASTs. First, the identified sequences were randomly blasted to the sequence data without indication of specific species. Second, we used random BLAST to the human genome and thirdly to the mycobacterium genome. We selected the proteins with highest homology with our peptide sequence.

### 2.7. Data Acquisition and Pre-Processing

Following the immunoreaction, the microarrays were scanned in an Axon Laboratories 4100 scanner (Palo Alto, CA, USA) using 532 and 647 nm lasers to produce a red (Alexa Fluor 647) and green (Alexa Fluor 532) composite image. Cy5 (red dye) labeled anti-human antibody was used to detect IgGs in human serum that were reactive to peptide clones, and a Cy3 (green dye) labeled antibody was used to detect the phage capsid protein [18]. Using the ImaGene 6.0 (Biodiscovery) image analysis software, the binding intensity of each peptide with IgGs in sera was expressed as *log_2_*(red/green) fluorescent intensities. These data were pre-processed using the limma package in the R language environment [19,21,22] and the normexp method was applied to correct the background [19,23]. Within array normalization was performed using the *LOESS* method [18,23,24]. The scale method was applied to normalize between arrays [23,24]. Intensity ratio of a clone in active TB samples divided by the same clone intensity ratio from healthy control samples were calculated to determine the fold change of a clone. 

### 2.8. Statistical Analyses

To detect differentially expressed antigens for TB, we applied a two-tailed *t-*test. In order to correct for multiple comparisons, we applied the false discovery rate (FDR) algorithm with a threshold of 0.01 FDR [25]. We identified 192 significant clones at 0.01 FDR. All significant clones were sorted in an increasing order. The top ten highly significant clones were considered as “classifier clones”. We randomly split the TB and healthy controls samples into: (i) training; (ii) test sets. Out of the 24 TB samples, 12 samples were randomly assigned to training set and 12 samples to testing set. The training and testing sets for the 45 healthy controls were randomly selected to 23 training and 22 test sets. A *t-*test was applied between TB-training samples versus healthy controls training samples. All 107 sarcoidosis samples were assigned to the testing set. To assess the performance of classifiers clones, we applied principal component analysis (PCA), agglomerative hierarchal clustering (HC), heatmap, and linear discriminant analysis (LDA). The LDA model was built on the training samples to predict TB samples from others (healthy controls and sarcoidosis) samples, and tested the classification model on the testing set (samples not used in the training set). We performed the classification on the classifiers clones. We applied principal component analysis (PCA), agglomerative hierarchal clustering (HC), and heatmap with all samples (training and testing) twice. Those analyses were first applied to all clones (1070 clones) and then with the highly significant 10 classifier clones. 

## 3. Results

### 3.1. Complex Sarcoidosis (CSL) Library Detects Unique Antigens in the Sera of Active Tuberculosis Patients

**Figure 1:**
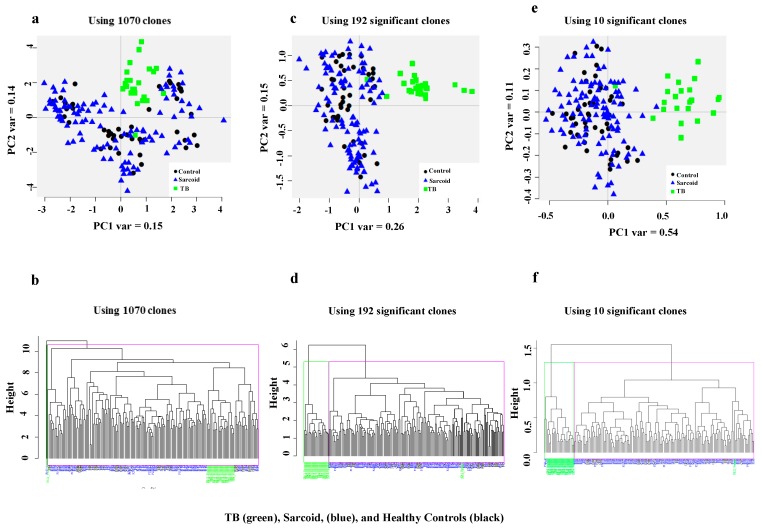
PCA and Hierarchal clustering. (**a**) PCA score plots along PC1 and PC2 generated with 1070 clones of three groups: (1) healthy control samples (black circles); (2) TB samples (green squares) and; (3) Sarcoidosis samples (blue triangle). Biomarker clusters along the PCA1 explain a variance of only 0.15, while the variance along PC2 was about 0.14. (**b**) The hierarchal clustering was applied on the healthy controls (black labels), TB patients (green labels) and sarcoidosis (blue labels) with 1070 clones. (**c**) PCA score plots along the PC1 and 2 results when applied on 192 TB clones. The PC1 explained 0.26 of variance, whereas PC2 explained 0.15 of variance. As shown, the TB samples are well separated from the healthy controls and sarcoidosis samples. (**d**) Hierarchal clustering using only the highly significant 192 TB clones. The blue and black clusters include sarcoidosis and healthy controls, the green cluster includes all the TB samples except one. (**e**) PCA score plots along PC1 and 2 generated with top 10 highly significant clones. The PC1 explained 0.54 of variance, whereas PC2 explained 0.11 of variance. (**f**) Hierarchal clustering using 10 top significant TB clones. This figure demonstrates better clustering with 192 TB clones and the highly significant 10 TB clones (panels c, d, e, and f) when compared the clustering using all clones (panels a and b).

A panel of potential antigens was randomly selected from two highly enriched pools of T7 phage cDNA libraries through biopanning of the CSL library [18,19]. The constructed microarray platform was immunoscreened with 176 sera (45 healthy controls, 24 smear-positive TB patients, and 107 sarcoidosis patients). The demographics of the study subjects are shown in Table 1. Following immunoreaction, the microarray data were pre-processed and then analyzed. First, we performed an unsupervised PCA using all 1070 clones with data from 176 study subjects. As shown in Figure 1A, several healthy controls and sarcoidosis patients clustered together with TB subjects. We also performed unsupervised hierarchical clustering with all 1070 clones on these 176 samples. We observed the magenta cluster has a mix of samples and lacks specific sub-clusters of TB samples (Figure 1B). Next, we applied a two-tailed *t-*test and identified 192 clones that were differentially expressed in sera of smear-positive TB as compared to sarcoidosis patients and healthy controls at the FDR < 0.01. To determine whether the selected 192 significant clones can improve the class separation of TB samples from healthy controls and sarcoidosis patients, we constructed a PCA plot. As shown in Figure 1C, there is a good separation of TB samples from sarcoidosis and healthy controls, in which twenty six percent of variance was along the PCA1. Similarly, when clustering algorithm was performed using 192 TB clones on all subjects, we observed a distinct hierarchical linkage clearly separating TB samples from healthy controls and sarcoidosis patients (Figure 1D). Furthermore, we constructed a PCA plot using 10 classifier clones that can differentiate TB patients from healthy controls and sarcoidosis patients. The result in Figure 1E shows a clear separation of TB samples from healthy controls and sarcoidosis patients. Fifty four percent of variance was explained along the PCA 1. Similarly, when the clustering algorithm was performed using 10 TB classifier clones, we observed a distinct hierarchical linkage separating the TB patients from others (Figure 1F).

**Figure 2:**
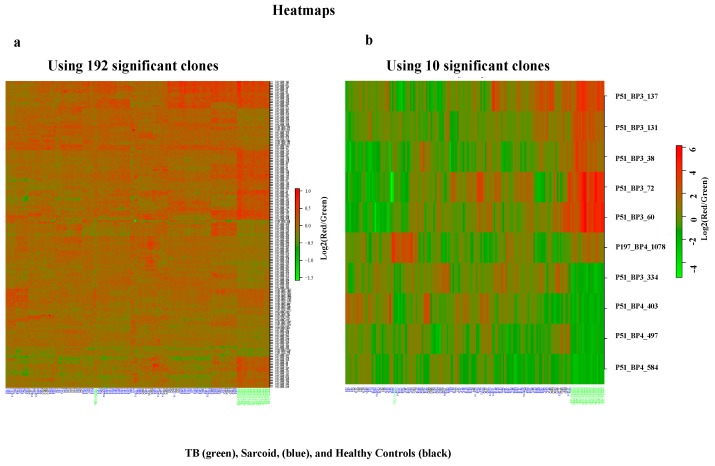
Heatmaps generated based on 192 clones and the 10 highly significant clones from the data of 176 study subjects (45 healthy controls, 24 with TB, and 107 with sarcoidosis) (**a**,**b**). Each row represents a clone, while each column represents a study subject. As shown in Figure 2, most of TB samples clustered to the right side of heatmap plots, while sarcoidosis samples and healthy controls clustered on the left side of the plot, indicating different expression profiles.

Figure 2A displays a heatmap plot of the distinct expression features of 192 TB clones among the study subjects. The heatmap using ten significant TB clones (classifiers) among study subjects is highlighted as a plot in Figure 2B.

**Figure 3:**
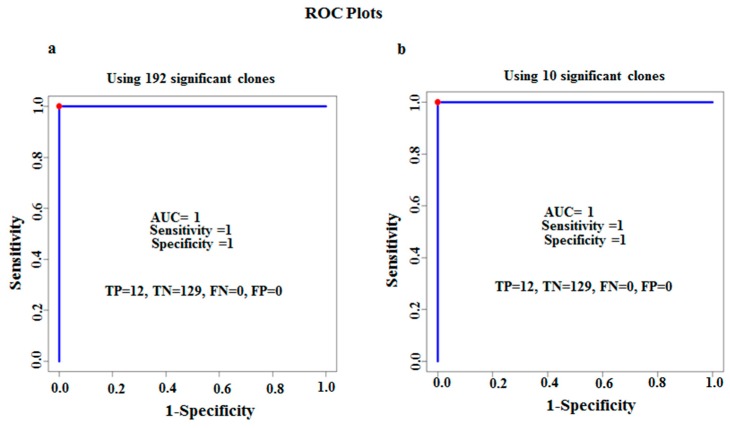
Classifiers to predict TB from healthy controls and sarcoidosis patients. (**a**) Performance of 192 clones on test set. (**b**) Performance of the top 10 classifier clones on test set. The ROC curves demonstrate excellent classification performance with AUC of 1 with sensitivity of 1 and specificity of 1.

Furthermore, we applied the classifier model and calculated the AUC values using 192 TB clones on testing sets. As shown in Figure 3A, the AUC under the ROC using 192 clones was one with no false positive and no false negative prediction. Next, we applied the classifier model on the test set (12 TB patients, 107 sarcoidosis patients, and 22 healthy controls) using 10 classifier clones. Figure 3B, shows that despite reduction of clones to 10, the AUC under the ROC remained one, again with no false positive or false negative class labeling. These suggest robust classifier performance. 

### 3.2. Characterization of Ten TB Classifiers

**Figure 4:**
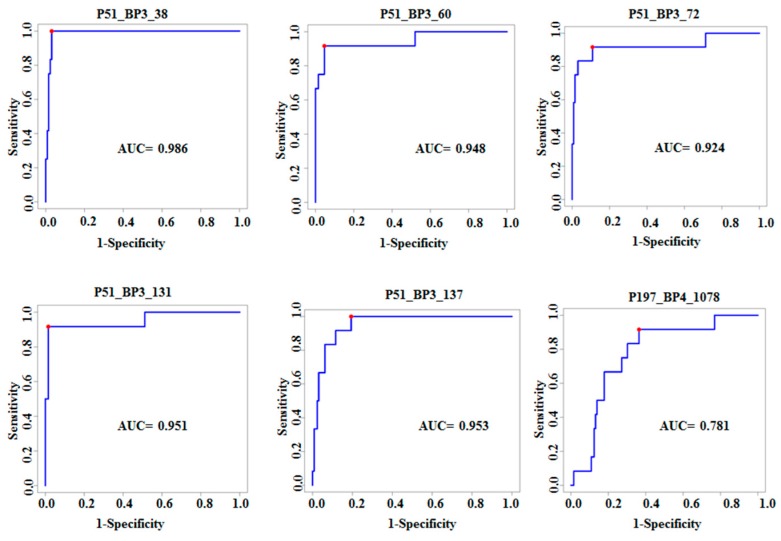
ROCs for top 6 significant clones that are increased in TB sera compared to healthy controls and sarcoidosis.

**Figure 5:**
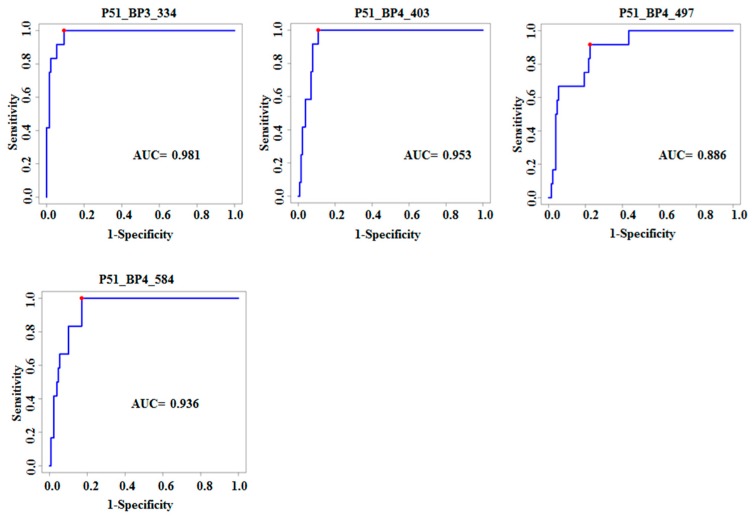
ROC for the top 4 significant clones that are decreased in TB sera compared to healthy controls and sarcoidosis. This figure demonstrates reasonable classification performance when classification was applied to one clone.

Based on the results of training and test sets, we characterized the top 10 highly performing active TB clones through sequencing. After obtaining the sequences of clones, the Expasy program was used to translate the cDNA sequences to peptide/protein sequences [18,19]. Protein blast using algorithms of the BLAST program were applied to identify the highest homology to identified peptides [18,19]. The identified clones were blasted with human and MTB genomes and then selected those specific peptide sequences with the highest homology of amino acids sequence. All top 10 clones have the highest homology with TB sequences. Additionally, we compared these results with corresponding nucleotide sequences using nucleotide BLAST and determined the predicted amino acids in frame with T7 phage 10B gene capsid proteins. All of the 10 classifier clones are coded by the inserted gene fragments leading to out-of-frame peptides, therefore meeting the criteria of mimotopes [26] (Table 2). As sera of active TB patients reacted with these out-of-frame peptides, it is likely that these TB clones are produced as a result of altered reading frames or alternative splicing, as described in previous studies [18,19,26]. Full length of peptides and genes of the ten classifiers clones are shown in Table S1. Table 2 shows the 10 most significant TB antigens, gene names, sensitivity, specificity, and FDR adjusted *p*-values. Figure 4 shows the ROC curves for six clones that are increased in TB, while Figure 5 shows ROC curves for four clones decreased in TB. 

## 4. Discussion

Standard methods to diagnose TB and to monitor response to treatment rely on sputum microscopy and culture. The current CDC/NIH roadmap emphasizes the need for development of new TB biomarkers as alternative methods [2]. Recently, a tremendous effort has been put forward elucidating the antibody responses to MTB antigens, which has implications for the development of new antigens to diagnose and monitor successful treatment, as well as to develop effective vaccination [27]. Most other studies searching for TB antigens have identified unspecific markers primarily involving host response such as C-reactive protein, serum amyloid A and other non-specific markers [28,29]. 

In view of this background, we hypothesized complex library derived from sarcoidosis tissue may harbor degradation products of MTB antigens and these antigens can be used as a bait to specifically and selectively bind to antibodies present in sera from active TB subjects. Our microarray platform identified 10 highly significant TB clones that can discriminate TB patients from healthy controls and sarcoidosis patients. All of these clones are TB specific and related to bacterial growth of *M. Tuberculosis* and its metabolism (Table 2). We sequenced the top 10 highly significant clones for TB and identified homologies in a public database. The range in length of identified peptides for TB antigens was between 6–23 amino acids (AA). Among the 10 TB specific phage peptides, six out-of-frame peptides were increased in sera of active TB patients (Table 2). One of the highly sensitive and specific peptide antigens (P51_BP3_38) identified in sera from active TB subjects is polyketide synthase (PKS). There are about 24 PKS encoding genes in *M. Tuberculosis*. This is an essential enzyme for mycolic acid formation [30]. The cell envelope of *M. Tuberculosis* is distinctive and associated with its pathogenicity and resistance. Mycolic acid is a long chain fatty acid found in the cell wall of *M. Tuberculosis* and this compound constitutes major strategic elements of the protective coat surrounding the tubercle bacillus [30]. Moreover, the cyclopropane ring of mycolic acid protects the bacteria from oxidative stress [31]. Another identified peptide antigen (P51_BP3_60) highly reactive to sera of MTB patients was hydrolase. *M. Tuberculosis* secretes hydrolases that have lipase activity and catalyzes lipid hydrolysis. They are responsible for the degradation of host lipid material [31]. It has become clear that in vivo MTB prefers to consume fatty acids and lipids over carbohydrates. Tubercle bacillus utilizes the host derived lipids/fatty acids as nutrients for prolonged persistence in a hypoxic environment [31].

Ferredoxin is another antigen (P51_BP3_72) significantly increased in sera of MTB patients. Ferredoxins are acidic, soluble iron–sulfur proteins. They act as redox partner for the cytochrome P450 enzyme (CYP51B). The *M. Tuberculosis* genome contains 20 CYPs. They are involved in metabolic processes such as epoxidation, sulfoxidation, and hydroxylation. *M. Tuberculosis*’s CYPs and their redox partners such as ferredoxin are essential for pathogen viability [32]. Another important MTB specific peptide antigen (P197_BP4_1078) belongs to the signal peptidase I (SPase I) enzyme. This is a membrane-bound endopeptidase responsible for cleavage of signal peptides of secreted proteins [33]. SPase I is an attractive target for the development of novel anti-tuberculosis treatments because first, it is essential for survival in all bacterial species; and secondly, bacterial SPase I is distinctively different from eukaryotic SPase I. Similarly, peptide antigen (P51_BP3_131) dihydroxyacid dehydratase (DHAD), which is involved in the growth of *Mycobacterium* is significantly increased. It is a key enzyme involved in branched-chain amino acid synthesis and also catalyzes the synthesis of 2-ketoacids from dihydroxyacids. It has been shown that the downregulation of this enzyme inhibits the growth of *M. Tuberculosis* in vitro and in mice model of TB infection [34]. The peptide antigen (P51_BP3_137) increased in MTB belongs to transketolase (Tkt) enzyme. This enzyme catalyzes the synthesis of ribose-5-phosphate (R5P) from the intermediates of the oxidative pentose phosphate pathway. Studies have shown that the depletion of Tkt using RNA silencing and protein degradation systems arrested the growth of *M. Tuberculosis* in vitro. The studies further demonstrated, using an ex vivo model of TB transfection in THP-1 cells, that Tkt-depleted bacteria showed less virulence as compared to wild type bacilli, confirming the essentiality of this enzyme in intracellular growth [35]. The three peptide antigens (transketolase, ferredoxin, and dihydroxy acid dehydratase) identified with the present study were also identified in our previous published study using sera from culture positive but smear negative patients [18]. These results clearly demonstrate the importance of these peptide antigens in TB. Among ten mimotopes, we found four with decreased expression in TB patients (Table 2). Interestingly, one of these four peptides with higher sensitivity and specificity (P51_BP3_334), belongs to repressor transcriptional regulators such as TetR [36]. TetR is involved in the regulation of antibiotic resistance and controls the expression of membrane-associated proteins involved in antibiotic resistance [37,38].

In this study, we have identified 10 highly significant clones from the sera of smear positive TB patients. These identified clones are mostly involved in the growth and virulence of *M. Tuberculosis.* Most of these clones have high specificity and sensitivity. Previous studies using a combination of ESAT-6 and CFP10 antigens, which are two *Mycobacterium tuberculosis*-specific antigens, to diagnose TB provided a sensitivity of 73% and 93% of specificity [39,40]. While studies in countries with higher TB prevalence has shown even lower sensitivity and specificity using various antigens including ESAT-6 and CFP10 [41]. Interestingly, Drake and et.al showed that higher percentage of sarcoidosis subjects (16/26) exhibit immunoreactivity to ESAT-6 and katG [9]. Our results appear to have a higher sensitivity and specificity as compared with those studies. One limitation of our study is that we did not include infected subjects with non-tuberculous mycobacteria. Although among the control group, 16 Asian subjects had BCG vaccination and 6 had positive quantiferon gold tests, we did not have enough power to detect possible differences between subjects with latent TB and active TB infection. Larger studies using sera from diverse populations including, subjects with non-tuberculous mycobacterial infection, latent TB infection and after BCG vaccination need to further validate the sensitivity and specificity of our classifiers. 

We detected these novel antigens using a heterologous library derived from sarcoidosis subjects. Lungs are highly exposed to numerous bacteria and our library is predominantly derived from sarcoidosis BAL cells and WBCs containing diverse immune cells, including macrophages that were exposed to various pathogens. We postulate that the CSL represents a segment of the lung microbe containing diverse antigens for TB, sarcoidosis, and cystic fibrosis [18,19,20]. 

There are various applications of a phage display. In the current work, we used a phage display for the discovery of TB biomarkers. The same system can be applied to identify novel markers for multi-drug resistance in TB, which is becoming a major issue in TB treatment. Additionally, phage displays can be used for the development of specific targeted therapies [42]. The phage display technology and immunoscreening has utilities not only in identifying diagnostic biomarkers, but also may enable us to develop a novel targeted therapy utilizing the peptide sequences (mimotopes) as vehicles to deliver specific drugs. The identified sequences can be used to develop peptide/protein-coated magnetic nanoparticles for clinical testing or for applications in drug delivery [43]. Additionally, this technology might enable us to discover unknown epitopes targeting specific bacterial antigens leading to immunogenicity and antibody production in TB subjects, as well as providing us with a better understanding of host immune defenses in TB subjects. For instance, TB sera were less reactive to some of the identified clones (TetR, menD, CobN, and OplA), these clones are less likely to be used for diagnostic purposes. However, these clones can be used to develop new vaccine and to boost the immunity against TB infection. Furthermore, this microarray platform can be hybridized to detect IgA in sputum of TB patients that may have clinical values. Moreover, antibody detection in the sera of patients has a potential value in clinical practice, as it is non-invasive and requires a minimal amount of blood or other bodily fluids.

The lack of sensitivity and specificity and cross-reactivity of biomarkers with other diseases dampened the enthusiasm in TB biomarker discovery studies. However, our study shows excellent sensitivity and specificity, not only as compared to healthy controls but also to another granulomatous disease. Other studies using gene expression profiling between TB and sarcoidosis found 94% similarities [44,45]. Our system has the advantage of detecting TB clones with high sensitivity and specificity and is based on an immune reaction rather than gene expression. The detection of this immune reaction, in form of antibodies, relies on a complex interaction between antigen presenting cells, T cells and B cells that leads to a specific antibody production in response to a TB infection. Highly specific biomarkers may have a potential role as candidate antigens in the development of novel vaccination for TB or for multidrug resistant bacterial infections. 

## Figures and Tables

**Table 1:** Subjects demographics.

Characteristic	Control Subjects	Sarcoidosis Subjects	TB Subjects
Age (Mean ± SEM)	40.3 ± 7.5	30.6 ± 11.8	40.5 ± 8.5
Gender, N (%)			
Male	11 (25)	27 (25)	18 (64)
Female	34 (75)	80 (75)	10 (36)
Race, N (%)			
African American	31 (69)	95 (89)	
African	-		4 (25)
Caucasian	-	12 (11)	
Asians	14 (31)		20 (75)
BMI (Mean ± SEM)	27 ± 3.8	28 ± 10.5	28 ± 6.9
Organ involvement			
Neuro-ophthalmologic	NA	31 (29)	-
Lung	NA	101 (94)	24 (100)
Skin	NA	46 (43)	-
Multiorgan	NA	65 (61)	-
PPD ^a^	NA	Negative	-
TB smear ^b^	NA	Negative	Positive

NA = not applicable; ^a^ PPD = Mantoux test (purified protein derivative); ^b^ TB Smear obtained from sputum.

**Table 2:** 10 Top Significant TB Clones.

Clone	Increased in Tuberculosis (TB)	Gene Name	*p* Value	FDR Corrected *p* Value	AUC 95% CI	Sensitivity, 95% CI	Specificity, 95% CI
**P51_BP3_38**	**Polyketide synthase**	**Pks13** **Rv3800c**	**4.7 × 10^−7^**	**2.79 × 10^−5^**	**0.98**	**1**	**0.97**
**P51_BP3_60**	**Hydrolase**	**Rv1723**	**1.62 × 10^−8^**	**3.46 × 10^−6^**	**0.95**	**0.92**	**0.95**
**P51_BP3_72**	**Ferredoxin**	**fdxA** **Rv2007c**	**1.36 × 10^−9^**	**7.27 × 10^−7^**	**0.92**	**0.91**	**0.89**
**P51_BP3_131**	**Dihydroxy acid dehydratase**	**ilvD** **Rv0189c**	**2.15 × 10^−8^**	**3.84 × 10^−6^**	**0.95**	**0.92**	**0.98**
**P51_BP3_137**	**Transketolase**	**TKT** **Rv1449c**	**7.14 × 10^−8^**	**9.72 × 10^−6^**	**0.95**	**1**	**0.81**
**P197_BP4_1078**	**Signal peptidase**	**lepB** **Rv2903**	**1.44 × 10^−7^**	**1.36 × 10^−5^**	**0.78**	**0.92**	**0.64**
	**Decreased in Tuberculosis (TB)**						
**P51_BP3_334**	**TetR family transcriptional regulator**	**MRA_2532**	**4.02 × 10^−10^**	**4.3 × 10^−7^**	**0.98**	**1**	**0.91**
**P51_BP4_403**	**Menaquinone biosynthesis protein**	**menD** **Rv0555**	**7.27 × 10^−8^**	**9.71 × 10^−6^**	**0.95**	**1**	**0.87**
**P51_BP4_497**	**Cobalamin biosynthesis protein**	**CobN** **Rv2062c**	**1.11 × 10^−8^**	**2.96 × 10^−6^**	**0.88**	**0.92**	**0.78**
**P51_BP4_584**	**5-oxoprolinase**	**OplA** **Rv0266c**	**5.82 × 10^−9^**	**2.10 × 10^−6^**	**0.94**	**1**	**0.83**

## Supplementary Materials

The following are available online at http://www.mdpi.com/1999-4915/10/7/375/s1, Table S1: Full length of sequence analysis of top 10 TB phage clones using NCBI BLAST.
