# Supplementary material for: Novel T7 Phage Display Library Detects Classifiers for Active Mycobacterium Tuberculosis Infection
Source: Viruses. 2018 Jul 19;10(7):375. doi: 10.3390/v10070375 (PMC6070804; doi:10.3390/v10070375)
Supplement: Supplementary file 1 [file viruses-10-00375-s001.pdf]

Table S1. Full length of sequence analysis of top 10 TB phage clones using NCBI BLAST

Rank	Clone and peptide size	Peptide sequences of mimotopes in-frame with T7 10B gene	Description of the sequences that mimotopes mimic	Region of similarity of peptide
**1**	P51_BP3_38 (23aa)	S A C L Q S L R T Q L L T W A L V G D V G Q P	polyketide synthase, partial [Mycobacterium tuberculosis] Sequence ID: WP_072515331.1	Id=8/9(89%) Gaps=1/9(11%) Length=894<br>Query 2 ACLQSLRTQ 10<br>AC QSLRTQ<br>Sbjct 283 AC-QSLRTQ 290
**2**	P51_BP3_60 (17aa)	S S E R N G Q F P W P L K M F L T	hydrolase [Mycobacterium tuberculosis] Sequence ID: CFG62212.1	Id=7/10 (70%) Gaps=0/10(0%) Length=280<br>Query 7 QFPWPLKMFL 16<br>Q WPL MFL<br>Sbjct 46 QLRWPLRMFL 55
**3**	P51_BP3_72 (9aa)	I Q H Q H L G Q I	ferredoxin [Mycobacterium tuberculosis] Sequence ID: ref|WP_003918358.1|	Id=6/6 (88%) Gaps=0/6 (0%) Length=105<br>Query 2 QHQHLG 7<br>QHQHLG<br>Sbjct 56 QHQHLG 61
**4**	P51_BP3_131 (14aa)	L Q A C F P Q I L R G S L A	dihydroxy-acid dehydratase IlvD [Mycobacterium tuberculosis TKK-01-0051] Sequence ID: gb|KBZ68064.1|	Id=7/7(100%) Gaps=0/7(0%) Length=570<br>Query 8 ILRGSLA 14<br>ILRGSLA<br>Sbjct 395 ILRGSLA 401
**5**	P51_BP3_137 (17aa)	D L S S E V A T H Q P I I A C L P	transketolase [Mycobacterium tuberculosis] Sequence ID: ref|WP_016721353.1|	Id=5/5(100%) Gaps=0/10(0%) Length=651<br>Query 8 THQPI 12<br>THQPI<br>Sbjct 453THQPI 457
**6**	P51_BP3_334 (18aa)	L P W S S G V C D I D L K W M V F S	TetR family transcriptional regulator [Mycobacterium tuberculosis] Sequence ID: ref|WP_015628739.1|	Id=6/7 (86%) Gaps=0/7 (0%) Length=265<br>Query 6 GVCDIDL 12<br>GVCD DL<br>Sbjct 250 GVCDTDL 256
**7**	P51_BP4_403 (11aa)	D C V V M T S G T L L	menaquinone biosynthesis protein MenD [Mycobacterium tuberculosis]	Id=7/8 (88%) Gaps=0/8 (0%) Length=486<br>Query 2 CVVMTSGT 9<br>CV MTSGT<br>Sbjct 74 CVAMTSGT 81

**8**	P51_BP4_497 (6aa)	S G K N Y L	Sequence ID: CKO35590.1 cobalamin biosynthesis protein CobN [Mycobacterium tuberculosis] Sequence ID: ref|WP_03165914 7.1|	Id=5/5 (100%) Gaps=0/5 (0%) Length=1174<br>Query 1 SGKNY 5<br>SGKNY<br>Sbjct 22 SGKNY 26
**9**	P51_BP4_584 (18aa)	S V E R F I N E S N D S M N N A T F	5-oxoprolinase [Mycobacterium tuberculosis] Sequence ID: ref|WP_00390632 1.1|	Id=6/11(55%) Gaps=0/11(0%) Length=383<br>Query 8 ESNDSMNNATF 18<br>E MNN TF<br>Sbjct 218 EGSGTMNNVTF 228
**10**	P197_BP4_107 8 (12aa)	H E Y A Q A S E E A R L	Signal peptidase I LepB [Mycobacterium tuberculosis] Sequence ID: CNI35074.1	Id=7/10(70%) Gaps=0/10(0%) Length=384<br>Query 3 YAQASEEARL 12<br>Y QA EE RL<br>Sbjct 153 YSQAQEEGRL 162
